# Peptaibol Analogs Show Potent Antibacterial Activity against Multidrug Resistant Opportunistic Pathogens

**DOI:** 10.3390/ijms24097997

**Published:** 2023-04-28

**Authors:** Chiara Dalla Torre, Filomena Sannio, Mattia Battistella, Jean-Denis Docquier, Marta De Zotti

**Affiliations:** 1 Department of Chemical Sciences, University of Padova, Via Marzolo 1, I-35131 Padova, Italy; chiara.dallatorre.2@studenti.unipd.it (C.D.T.); mattia.battistella.1@studenti.unipd.it (M.B.); 2 Dipartimento di Biotecnologie Mediche, University of Siena, Viale Bracci 16, I-53100 Siena, Italy; filomena.sannio@unisi.it (F.S.); jddocquier@unisi.it (J.-D.D.); 3 Lead Discovery Siena s.r.l., Via Fiorentina 1, I-53100 Siena, Italy; 4 Laboratoire de Bactériologie Moléculaire, Centre d’Ingénierie des Protéines—UR InBioS, University of Liège, Allée du Six Août 11, B-4000 Liège, Belgium

**Keywords:** multidrug-resistant pathogens, peptides, Aib, peptaibol, helical conformation, *Acinetobacter baumannii*, *Pseudomonas aeruginosa*

## Abstract

New classes of antibacterial drugs are urgently needed to address the global issue of antibiotic resistance. In this context, peptaibols are promising membrane-active peptides since they are not involved in innate immunity and their antimicrobial activity does not involve specific cellular targets, therefore reducing the chance of bacterial resistance development. Trichogin GA IV is a nonhemolytic, natural, short-length peptaibol active against Gram-positive bacteria and resistant to proteolysis. In this work, we report on the antibacterial activity of cationic trichogin analogs. Several peptides appear non-hemolytic and strongly active against many clinically relevant bacterial species, including antibiotic-resistant clinical isolates, such as *Staphylococcus aureus*, *Acinetobacter baumannii*, and extensively drug-resistant *Pseudomonas aeruginosa*, against which there are only a limited number of antibiotics under development. Our results further highlight how the modification of natural peptides is a valuable strategy for obtaining improved antibacterial agents with potential therapeutic applications.

## 1. Introduction

In the context of the current antimicrobial resistance (AMR) crisis [1,2], antimicrobial peptides (AMPs, [3]) are rapidly emerging as a promising class of compounds to combat human pathogenic bacteria [4,5]. As part of well-established peptide antibiotics, such as the glycopeptide vancomycin [6] and the cationic cyclic polymyxins [7], several bioactive peptides targeting different cellular components have been identified and studied as novel antibacterial drugs. For instance, apidaecin (Api137) is a peptide extracted from honeybees and targets the ribosome [8,9]. Murepavidin is a peptidomimetic that interacts with the bacterial lipopolysaccharide transport protein LptD [10,11]. The exploitation by this type of AMPs of specific DNA-encoded bacterial macromolecules (i.e., the ribosome or enzymes involved in essential metabolic pathways) as targets, however, may lead to the development of bacterial resistance, potentially occurring through the accumulation of bacterial mutations. Alternative peptide-based therapeutics are required, especially against multidrug-resistant opportunistic pathogens such as *Pseudomonas aeruginosa* and *Acinetobacter baumannii*, which account for a significant number of infections in immunocompromised patients [12]. In this framework, the ability of several AMPs to act against the bacterial membrane is strategic [13]. On the other hand, innate immunity, which represents the first defense line of higher organisms, relies on membrane-active AMPs [14], and they are even part of the human defense system [15]. Therefore, concerns about the therapeutic use of such host defense peptides that might lead to the evolution of resistance were reasonably raised [16,17]. Moreover, despite the fact that bacteria in general display a lower propensity to develop resistance to AMPs than conventional small-molecule antibiotics, evidence of bacterial resistance to polymyxins and other membrane-active peptides has been reported in the literature [7]. Bacteria can develop resistance to AMPs by altering their anionic membrane lipopolysaccharides, which represent natural targets for cationic AMPs, or by the production of bacterial proteases able to neutralize them [18]. In this context, the use of nonribosomally produced AMPs is highly recommended since they are intrinsically proteolysis-resistant [19]. Peptaibols are naturally occurring, nonribosomal peptides endowed with antimicrobial activity [20,21]. Their mechanism of action is based on peptide-membrane interactions through their well-defined helical conformation [22,23]. Their sequence comprises a C-terminal 1,2-aminoalcohol and several C^α^-tetrasubstituted residues, the simplest and most common of which is α-aminoisobutyric acid, or Aib (α-methylalanine), a helix-inducer residue [24], hence the name pept*Aib*-ol [25]. In the current emergency due to the rapid onset of antimicrobial drug resistance, peptaibols thus represent a great opportunity because (i) they are nonribosomally produced by fungi as part of their defense mechanism against other microorganisms; (ii) they are not part of multicellular organisms peptide-based defense weapons; (iii) they act against phospholipid membranes and lack a specific target in bacteria; and (iv) they are not recognized by proteolytic enzymes, overcoming the known limitations of peptides as orally available active agents and reducing the chance of bacterial resistance through the enzymatic mechanism. Trichogin GA IV [26,27] is a short-length peptaibol [28] produced by *Trichoderma longibrachiatum*, an ubiquitous fungus of the genus *Trichoderma*, used in organic farming [29,30]. Its sequence is reported in Table 1. It is active against Gram-positive bacteria [31] and is non-hemolytic [31]. Several trichogin analogs are endowed with plant protection properties against phytopathogens, especially those targeting grapevines, such as *Botrytis cinerea* [32] and *Plasmopara viticola* [33]. Trichogin GA IV and some of its analogs are also toxic against human cancer cells [34,35] while others are completely non-toxic [36]. Recently, results against phytopathogenic bacteria confirmed that cationic trichogin analogs are also promising antimicrobial compounds [37]. In this article, we report the results of our in-depth investigation on the antibacterial activity of various trichogin analogs against clinically relevant Gram-positive and Gram-negative bacterial strains, including the WHO “critical priority” pathogens [38]. A structure–activity relationship study on newly synthesized and characterized ultrashort trichogin analogs that allowed us to confirm that a stable helical conformation is paramount to the antimicrobial action is also described.

## 2. Results

### 2.1. Peptide Synthesis

Trichogin analogs were produced by solid-phase peptide synthesis (SPPS). The synthesis and characterization of peptides 0–11 (Table 1) have already been reported [32,37,39]. In the analogs, Lys residues replaced one or more Gly residues in the native sequence, with the aim of enhancing both the water solubility and the amphipathicity of the native helical structure [40]. With our sequence modifications, we aim to expand the antimicrobial spectrum of action of native peptaibol. Some cationic trichogin analogs have already been reported to have an increased antimicrobial action against phytopathogenic and human-targeting bacteria than the parent peptide [37,41]. New, ultrashort sequences 12–18 (Table 1) were designed and produced by SPPS following a green chemistry protocol, using an ethyl acetate/dimethylsulfoxide (DMSO) mixture as solvent [42]. The peptides were obtained with high yield (54–65%) and crude purity and further purified to >91% by medium-pressure liquid chromatography (Isolera Prime system, Biotage, Uppsala, Sweden). The peptides were characterized by high-pressure liquid chromatography (HPLC) and high-resolution mass spectrometry (HRMS). HPLC profiles and HRMS spectra of the new sequences are reported in the Supporting Material.

### 2.2. Antibacterial Activity and Hemolysis

The antibacterial activity of the peptides was initially assessed on a panel of representative Gram-positive and Gram-negative organisms of high clinical relevance and commonly responsible for bacterial infections in both animals and humans. *Bacillus subtilis* ATCC 6633 (Bsu) was also included as a rod-shaped model Gram-positive organism. This first panel comprised: *Enterococcus faecalis* ATCC 29212 (Efa); *Streptococcus pyogenes* ATCC 12344 (Spy); and *Staphylococcus aureus* ATCC 25923 (Sau); Gram-negative: *Escherichia coli* CCUGT (Eco), *Klebsiella pneumoniae* ATCC 13833 (Kpn), *Acinetobacter baumannii* ATCC 17978 (Aba), and *Pseudomonas aeruginosa* ATCC 27853 (Pae) (Table 2). Both the minimum inhibitory concentration (MIC, Table 2) and the minimum bactericidal concentration (MBC, Table S1, Supporting Material) were determined. Peptides 1–12 showed potent antibacterial activity against Gram-positive bacteria, as well as *A. baumannii* and *P. aeruginosa*, which represent WHO “critical priority” organisms for the development of new antibiotics [1]. The peptide analogs with the lowest MIC (2 µg/mL) were 1, 4, 7, and 11, while peptide 5 showed the broadest spectrum of activity, with MIC values of 4–8 µg/mL against six of the eight bacterial strains tested and 16 µg/mL against the other two. Notably, some of those peptides also showed very low MBC values. For example, peptide 4 was effective against *B. subtilis* (Bsu), *S. pyogenes* (Spy), and *A. baumannii*. (Aba) with MIC and MBC = 2 µg/mL, while its MIC and MBC against *E. faecalis* (Efa) were 2 and 4 µg/mL, respectively (Table 2 and Table S1, Supporting Material). Peptide 7 was found to be effective against all tested bacterial strains, with MIC and MBC ranging from 2–16 µg/mL and 8–16 µg/mL, respectively. Both peptides 4 and 7 are also non-hemolytic (Table 2). We compared our results with those reported in a previous study for trichogin GA IV and five of its analogs, including peptides 1, 4, 5, and 6 of the present study, against *E. coli* ATCC25922, *S. aureus* ATCCBAA-44, *P. aeruginosa* ATCC25668, and *Staphylococcus epidermidis* ATCC700565 [43]. In that previous study, the well-known antibacterial activity of native trichogin GA IV against Gram-positive bacteria was questioned [43]. Here, we unambiguously confirm the antibacterial activity of trichogin against three of the four Gram-positive bacterial strains tested. Trichogin GA IV is also known to be inactive against Gram-negative bacteria, as confirmed by our results. Moreover, cationic trichogin analogs, designed to broaden the spectrum of their antimicrobial action, were indeed able to effectively kill Gram-negative bacteria. Peptides 1, 4, 4b, 5, 5b, 7, 9, 10, and 11 showed MIC values between 2 and 8 μg/mL (corresponding to 1.8–7.2 μM) against *A. baumannii*, and some were also bactericidal at the MIC, namely peptides 4, 11, 4b (MIC and MBC, 2, 2, and 4 μg/mL, respectively) and peptide 9 (MIC and MBC, 8 μg/mL). On the contrary, ultrashort peptides 13–18 appeared to have no significant antibacterial activity. We hypothesized that this result is due to the lack of a defined helical conformation and therefore performed a conformational analysis (see below). The behavior of the 15 ultrashort peptides is particularly notable. Since its molecular weight (MW: 582.44 g/mol) is approximately half that of full-length analogs 1–11 (average MW: 1220 g/mol), its MIC was actually between 8 and 32 µM for all bacterial strains tested. On the other hand, it also showed measurable hemolytic activity (Table 2); therefore, we did not select it for further studies. Nevertheless, its simple and short sequence (comprising only one Aib and three protein amino acid residues) makes it a good starting point for the development of less-expensive and broad-spectrum antibacterial peptides.

To assess the selectivity of the peptides, their hemolytic activity was tested by spotting 50 µg of each peptide on a blood agar plate, resulting in a final peptide concentration much higher than the MIC. Hemolysis zones were visually determined and measured. Interestingly, there was no direct correlation between antibacterial activity and hemolysis. Most of the peptides (17 out of 24) were found non-hemolytic, showing a total absence or an undefined inhibition halo below the detection level (Table 2, Figure S13, Supporting Material). For two additional peptides (6 and 15), only a small hemolysis zone (diameter 4–5 mm) was visible, as compared to that of the positive control (Triton X-100, 13 mm).

The antibacterial activity of the most active peptide analogs was further evaluated against antibiotic-resistant clinical isolates (Table 3), including carbapenem-resistant and extensively drug-resistant (XDR) strains, which have become a global concern [44,45]. Data in Table 3 confirm the broad spectrum of antibacterial activity of peptides 5 and 7, which showed potent activity against both MRSA and carbapenemase-producing *A. baumannii* and *P. aeruginosa* clinical isolates [46]. Most interestingly, these peptides also showed potent bactericidal activity on the MDR *A. baumannii* clinical isolate SI-310 (Table 3), which is resistant to the last-resort, membrane-targeting peptide antibiotic colistin [7]. Our data prove that the mechanism of resistance to colistin does not result in cross-resistance to the peptides described in this study.

Notably, the MRSA strain was found to be more susceptible than the methicillin-susceptible reference strain (Table 2). Peptides remained strongly active against both MDR *A. baumannii* and *P. aeruginosa,* with MIC and MBC values between 2 and 4 µg/mL for most of them. These two bacterial species are considered of great interest and strongly epidemiologically representative in relation to pan-drug resistance evolution. In particular, peptides 5, 5b, 7, and 11 were found to be active even against XDR *P. aeruginosa* strain VR-143/97, with a MIC value of 8 µg/mL for all. In summary, the peptides showed promising activity on both MDR/XDR *A. baumannii* and *P. aeruginosa*, and very low hemolytic activity.

### 2.3. Structure-Activity Relationship

The mechanism of antimicrobial action of peptaibols is heavily based on their helical conformation [47,48]. The lack of antibacterial activity shown by the new ultrashort sequences (13–18, Table 1) prompted us to investigate their conformational preferences in solution by circular dichroism (CD), exploring different experimental conditions. The aqueous solution is useful to assess the stability of the helix, while the micelles of sodium dodecyl sulfate (SDS) 100 mM in water are a membrane-mimicking environment, which promotes the onset of helical conformations [49]. Peptide-membrane interactions usually involve the formation of helical bundles, which can be either parallel or perpendicular to the membrane surface, eventually causing leakage [50]. Some of the cationic trichogin analogs described here have already been reported to adopt a mixed, right-handed 3_10_/α-helical structure in the presence of SDS micelles [41,51]. The CD spectra of unordered peptides are characterized by the presence of a negative maximum centered at approximately 150 nm and a positive maximum at about 215 nm [52]. In contrast, the CD profile for a peptide adopting a α-helical conformation features two negative maxima centered at approximately 208 and 222 nm and a positive maximum at approximately 190 nm, while for a 3_10_-helix, common for peptaibols, the first negative maximum shifts to about 205 nm [53].

**Figure 1:**
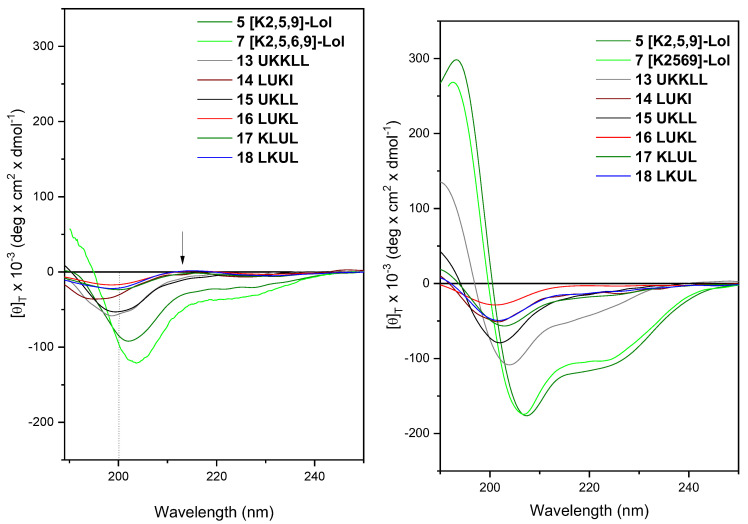
CD spectra of the most active sequences 5 and 7 (peptide concentration, 1 mM) and the ultrashort sequences 13–18 (peptide concentration, 5 mM) in water (**left**) and aqueous SDS 100 mM (**right**).

The CD study in water for the two most active sequences 5 and 7 (Figure 1) showed a 3_10-_helical conformation (negative maxima centered at about 205 and 222 nm). The CD profiles acquired for peptides 13–18 (Figure 1) show that the ultra-short, inactive peptides adopt a largely unordered 3D structure in water since their negative maximum falls below 200 nm and a positive one at about 215 nm is detectable (shown by an arrow, Figure 1). The stability of the peptide helical conformation in water can influence its antimicrobial activity; therefore, this observation might explain the very different efficacy of the analogs. It is noteworthy that the two ultrashort peptides that display some activity, namely 13 and 15, are the most structured. To verify whether the unordered structure of the ultrashort analogs was maintained in a membrane environment, we acquired the CD spectra in the presence of SDS micelles. The two full-length sequences 5 and 7 maintained the helical conformation, switching towards a more α-helical one (the first negative maximum centered at 208 nm). On the contrary, all ultrashort peptides displayed CD profiles closer to those of the helix, but the positions of the negative maxima did not achieve the canonical values for a 3D structure of helices (Figure 1). Again, the two most helical peptides are 13 and 15, which are also the most active among the ultrashort analogs, which supports the hypothesis that a certain degree of stability of the helical conformation is required for the trichogin analogs to exert antimicrobial activity.

## 3. Discussion

Peptaibol trichogin GA IV is a naturally occurring antibacterial peptide. In the present work, we assessed the activity of more than twenty of its analogs against several representative Gram-positive and Gram-negative bacteria. A variety of peptide sequence modifications were applied, including: (i) The substitution of Gly with Lys was chosen to improve water solubility, and because it improved peptide activity against phytopathogenic bacteria [37]. (ii) Sequence 8 contains an Aib-to-Api substitution. Api is a tetrasubstituted residue sharing the same conformational preferences for helical dihedral angles as Aib. Its insertion allowed us to assess the effect of the presence of a positive charge on the apolar face of the trichogin helical structure. This substitution improved peptide activity against Gram-positive bacteria but also increased peptide-induced hemolysis, suggesting that the low hemolytic activity of trichogin analogs is supported by the amphipathicity of their helical structure. (iii) The naturally occurring, C-terminal aminoalcohol Lol can be inserted in peptide sequences by SPPS using a preloaded 2-chlorotrytil resin. This resin is, however, very expensive, and its replacement with the cheaper rink amide resin, leading to a C-terminal amide, would be desirable. For this reason, we decided to evaluate the performance of C-terminal amide trichogin analogs. We therefore tested four pairs of Lol/Leu-NH_2_ analogs: 4/4b, 5/5b, 6/6b, and 10/10b. We did not find significant differences in their antibacterial activity, confirming that this C-terminal modification can be applied, thus reducing the cost of peptide production. (iv) The ultrashort analogs 14–18 were designed to reduce the peptide length as much as possible while maintaining the possibility for them to form β-turns (i.e., 1←3 intramolecularly H-bonded C_10_ pseudo-cycles). Aib-containing peptides are indeed able to form single β-turns, even when the sequence comprises only five amide bonds, as for the ultrashort sequences described here [54]. The presence of such an ordered structure for the tetrapeptides was, however, excluded by CD analysis, suggesting that the presence of only one Aib residue is not enough to support it. Interestingly, analog 15, with Aib at position 1, showed a CD profile closer to that of a helical conformation than its isomers 14, 16, 17, and 18, in which Aib is not at the N-terminus. Several trichogin analogs were found to be active against epidemiologically relevant MDR and XDR clinical isolates, including WHO “critical priority” pathogens for antibiotic research and development, with negligible hemolytic activity. The peptides were also active against XDR *P. aeruginosa*, against which only a limited number of antibiotics currently under development are active [55,56]. A structure–activity relationship built on CD analysis under different experimental conditions indicated that a well-defined helical conformation in water is needed for the peptides to exert their antimicrobial activity. Many trichogin analogs showed potent antimicrobial activity even against a colistin-resistant *A. baumannii* clinical isolate, demonstrating their ability to overcome some mechanisms of resistance involving modifications of the outer membrane. The results herein highlight the importance of exploring Aib-containing peptides, such as trichogin analogs, as a valuable source of highly active bactericidal compounds whose spectrum of activity fully addresses the current medical need, including MRSA and carbapenem-resistant Gram-negative opportunistic pathogens.

## 4. Materials and Methods

### 4.1. Peptide Synthesis

The SPPS of the C-terminal 1,2-aminoalcohol leucinol (Lol) sequences was carried out on a 2-chlorotrytil resin preloaded with Lol (Iris Biotech, Marktredwitz, Germany), while the C-terminal amide peptides were produced on a Rink amide resin. Fluorenylmethyloxycarbonyl (Fmoc) amino acids, active agents Oxyme pure and diisopropylcarbodiimide (DIC), reagents, and solvents were obtained by Sigma-Aldrich (Munich, Germany). Synthesis was performed by manual SPPS as described in [36], a part of Fmoc-deprotection, which has been performed by using a solution of piperidine 20% in ethyl acetate [57]. Coupling steps involving an Aib or Api residue as the nucleophile were repeated twice with fresh reagents. Peptides were detached from Rink amide resin by treatment with the cleavage cocktail: Trifluoroacetic acid (TFA) 95%, water 2.5%, and triisopropylsilane, 2.5%, for about three hours. On the other hand, a solution of hexafluoroisopropanol 30% in dichloromethane was used to detach still-protected peptides from the 2-chlorotrytil resin. The side-chain protecting group *tert*butyloxycarbonyl (Boc) was removed from Lys or Api residues of the C-terminal Lol-bearing peptides by treatment with a 3M methanolic solution of HCl for about 3–4 h. Precipitation from ethyl ether gave crude peptides with good yield (54–65%) and purity. The crude compounds were further purified by medium-pressure liquid chromatography on an Isolera Prime instrument (Biotage, Uppsala, Sweden). HPLC (eluants: A, H_2_O/CH_3_CN (9:1 *v*/*v*) + 0.05% TFA; B, CH_3_CN/H_2_O (9:1 *v*/*v*) + 0.05% TFA; flow rate 1 mL/min; λ = 216 nm) and electrospray ionization (ESI) high-resolution mass spectra (HRMS), obtained from a Waters Micromass instrument (Milford, MA, USA), are reported in the Supporting Material, together with the gradient conditions and calculated purity for each peptide.

### 4.2. Antibacterial Assays

The minimum inhibitory concentration (MIC) and the minimum bactericidal concentration (MBC) of the compounds were determined in triplicate using Mueller–Hinton broth (MHB). Although most peptides in Table 1 are water-soluble, the native sequences of trichogin GA IV and its analog 3 are not. Therefore, we decided to dissolve all peptides in DMSO to a final concentration of 25 mg/mL, for consistency. The bacteria inoculum was 5 × 10^5^ CFU/mL, as recommended by CLSI (Document M07-A10, 2015). MIC and MBC were recorded after 18 h of incubation at 35 °C. Reference-type strains, including representatives of both Gram-positive bacteria (*Bacillus subtilis* ATCC 6633, *Enterococcus faecalis* ATCC 29212, *Staphylococcus aureus* ATCC 25923, the methicillin-resistant *Staphylococcus aureus* ATCC 43300 (MRSA), *Streptococcus pyogenes* ATCC 12344) and Gram-negative bacteria (*Escherichia coli* CCUGT, *Klebsiella pneumoniae* ATCC 13833, *Pseudomonas aeruginosa* ATCC 27853, and *Acinetobacter baumannii* ATCC 17978), were acquired from ATCC or CCUG culture collections. Other antibiotic-resistant clinical isolates were present in our collection at the University Teaching Hospital (Azienda Ospedaliero-Universitaria Senese ‘Santa Maria alle Scotte’) in Siena, Tuscany, Italy. Specifically, MDR carbapenem-hydrolyzing class D β-lactamase-producing *Acinetobacter baumannii* and metallo-β-lactamase-producing *Pseudomonas aeruginosa* were used in this study (Table 4).

### 4.3. Hemolysis Assays

The hemolytic activity of the compounds was assessed using the method described by Bechlars et al. [58]. Briefly, 2 μL of each peptide solution in DMSO (25 mg/mL), corresponding to 50 μg, was spotted on the surface of a blood agar plate (Columbia CNA Agar, Becton Dickinson Italia S.p.A., Florence, Italy). The plate was then incubated at 35 ± 2 °C for 24 h and visually inspected for the presence of hemolysis zones (see Figure S13, Supplementary Material). The experiment was performed in triplicate. Table 2 reports the average value of the diameter of hemolysis zones measured in each individual experiment. Triton X-100 was used as a positive control (diameter of the hemolysis zone: 13 mm), while DMSO was used as a negative control.

### 4.4. Circular Dichroism

We acquired peptide CD profiles on a Jasco J-1500 spectropolarimeter (Tokyo, Japan) on fused quartz cells with a 1 mm path length (Hellma, Mühlheim, Germany). Ellipticity is expressed as [θ]_T_, total molar ellipticity (deg ∙ cm^2^ ∙ dmol^−1^). The temperature was kept at 25 °C. MilliQ water and a 100 mM SDS aqueous solution were used as solvents. The peptide concentration was between 1 and 5 mM.

## Figures and Tables

**Table 1:** Peptide sequences.

No.	Peptide Name ^a^	Peptide Sequence
0	Trichogin GA IV	1-Oct-Aib-Gly-Leu-Aib-Gly-Gly-Leu-Aib-Gly-Ile-Lol
1	[K^2,5^]-Lol	1-Oct-Aib-**Lys**-Leu-Aib-**Lys**-Gly-Leu-Aib-Gly-Ile-Lol
2	[K^2^]-NH_2_	1-Oct-Aib-**Lys**-Leu-Aib-Gly-Gly-Leu-Aib-Gly-Ile-**Leu-NH_2_**
3	[L^4^]-NH_2_	1-Oct-Aib-Gly-Leu-**Leu**-Gly-Gly-Leu-Aib-Gly-Ile-**Leu-NH_2_**
4	[K^5,6^]-Lol	1-Oct-Aib-Gly-Leu-Aib-**Lys**-**Lys**-Leu-Aib-Gly-Ile-Lol
4b	[K^5,6^]-NH_2_	1-Oct-Aib-Gly-Leu-Aib-**Lys**-**Lys**-Leu-Aib-Gly-Ile-**Leu-NH_2_**
5	[K^2,5,9^]-Lol	1-Oct-Aib-Gly-Leu-Aib-**Lys**-**Lys**-Leu-Aib-**Lys**-Ile-Lol
5b	[K^2,5,9^]-NH_2_	1-Oct-Aib-Gly-Leu-Aib-**Lys**-**Lys**-Leu-Aib-**Lys**-Ile-**Leu-NH_2_**
6	[K^5^,U^6^]-Lol	1-Oct-Aib-Gly-Leu-Aib-**Lys**-**Aib**-Leu-Aib-Gly-Ile-Lol
6b	[K^5^,U^6^]-NH_2_	1-Oct-Aib-Gly-Leu-Aib-**Lys**-**Aib**-Leu-Aib-Gly-Ile-**Leu-NH_2_**
7	[K^2,5,6,9^]-Lol	1-Oct-Aib-**Lys**-Leu-Aib-**Lys**-**Lys**-Leu-Aib-**Lys**-Ile-Lol
8	[Api^8^]-NH_2_	1-Oct-Aib-Gly-Leu-Aib-Gly-Gly-Leu-**Api**-Gly-Ile-**Leu-NH_2_**
9	[K^9^]-Lol	1-Oct-Aib-Gly-Leu-Aib-Gly-Gly-Leu-Aib-**Lys**-Ile-Lol
10	[K^6^]-Lol	1-Oct-Aib-Gly-Leu-Aib-Gly-**Lys**-Leu-Aib-Gly-Ile-Lol
10b	[K^6^]-NH_2_	1-Oct-Aib-Gly-Leu-Aib-Gly-**Lys**-Leu-Aib-Gly-Ile-**Leu-NH_2_**
11	[K^2,5,6^]-NH_2_	1-Oct-Aib-**Lys**-Leu-Aib-**Lys**-**Lys**-Leu-Aib-Gly-Ile-**Leu-NH_2_**
Ultrashort analogs		
12	[4-11]-NH_2_	1-Oct-Aib-Lys-Lys-Leu-Aib-Gly-Ile-Leu-NH_2_
13	UKKLL	1-Oct-Aib-Lys-Lys-Leu-Leu-NH_2_
14	LUKI	1-Oct-Leu-Aib-Lys-Ile-NH_2_
15	UKLL	1-Oct-Aib-Lys-Leu-Leu-NH_2_
16	LUKL	1-Oct-Leu-Aib-Lys-Leu-NH_2_
17	KLUL	1-Oct-Lys-Leu-Aib-Leu-NH_2_
18	LKUL	1-Oct-Leu-Lys-Aib-Leu-NH_2_

^a^ Amino acid substitutions with respect to the native sequence are highlighted in bold and indicated with the one-letter code in peptide names. Lol, leucinol; Aib, U, α-aminoisobutyric acid; Api, 4-aminopiperidin-4-carboxylic acid; 1-Oct, 1-octanoyl.

**Table 2:** In vitro antibacterial activity (Minimum inhibitory concentration, MIC) of trichogin GA IV and its analogs against reference Gram-positive and Gram-negative strains and hemolysis.

Peptide No. and Name	MIC (μg/mL) ^a^								Hemolysis (d, mm) ^b^
	Gram-Positive				Gram-Negative				
	Bsu	Efa	Spy	Sau	Eco	Kpn	Aba	Pae	
0 Tric-Lol	16	16	16	>128	>128	>128	>128	>128	-
1 [K^2,5^]-Lol	**4**	**2**	**2**	16	32	>128	**4**	16	-*
2 [K^2^]-NH_2_^c^	**4**	**4**	**4**	**8**	>128	64	16	>128	+ (8)
3 [Leu^4^]-NH_2_	>128	>128	>128	>128	>128	>128	>128	>128	-
4 [K^5,6^]-Lol	**2**	**2**	**2**	16	16	16	**2**	**8**	-*
4b [K^5,6^]-NH_2_	**2**	**4**	**2**	16	16	32	**4**	16	-*
5 [K^2,5,9^]-Lol	**8**	**4**	**4**	16	**8**	16	**4**	**8**	-*
5b [K^2,5,9^]-NH_2_	**2**	**2**	**2**	**8**	32	32	**4**	16	-*
6 [K^5^,U^6^]-Lol	**8**	**8**	**8**	**8**	>128	>128	>128	>128	+ (4)
6b [K^5^,U^6^]-NH_2_	128	128	**8**	128	>128	>128	>128	>128	-
7 [K^2,5,6,9^]-Lol	**2**	**4**	**2**	16	16	16	**2**	**8**	-*
8 [Api^8^]-NH_2_	32	16	16	>128	>128	>128	>128	>128	+ (7)
9 [K^9^]-Lol	**4**	**4**	16	16	>128	>128	**8**	>128	+ (10)
10 [K^6^]-Lol	**4**	**4**	**4**	**8**	64	16	16	>128	+ (11)
10b [K^6^]-NH_2_	**4**	**4**	**4**	32	>128	>128	16	>128	+ (10)
11 [K^2,5,6^]-NH_2_^c^	**2**	**4**	**4**	16	16	>128	**2**	**8**	-
12 [4-11]	**4**	32	16	16	16	64	32	64	-*
13 UKKLL	32	>128	>128	>128	>128	>128	>128	>128	-
14 LUKI	>128	>128	>128	>128	>128	>128	>128	>128	-
15 UKLL	16	32	16	64	64	64	64	64	+ (5)
16 LUKL	>128	>128	>128	>128	>128	>128	>128	>128	-
17 KLUL	>128	>128	>128	>128	>128	>128	>128	>128	-
18 LKUL	128	>128	128	>128	>128	>128	>128	>128	-

^a^ MIC values ≤ 8 µg/mL are highlighted in bold, with a green background; ^b^ -, no hemolysis observed; -*, limited hemolysis (hemolysis zone not defined); + hemolysis observed (d, diameter of hemolysis zone); ^c^ not completely soluble in Mueller–Hinton broth (MHB).

**Table 3:** In vitro antibacterial activity (MIC) of trichogin GA IV and its analogs against antibiotic-resistant clinical isolates of *S.aureus*, *A. baumannii*, and *P. aeruginosa*.

No.	MIC/MBC (μg/mL) ^a^							
	*S. aureus* *ATCC* 43300 (MRSA)	*A. baumannii* SI-12 (OXA-23)	*A. baumannii * SI-648 (OXA-23, OXA-51-like)	*A. baumannii* SI-310 (OXA-24)	*P. aeruginosa* VR-143/97 (VIM-1)	*P. aeruginosa* VA-182/00 (VIM-2)	*P. aeruginosa* 506/99 (VIM-2)	*P. aeruginosa* 101/1477 (IMP-1)
1	**2/2**	**4/8**	**8/16**	**4/4**	32/32	16/16	16/32	**8/16**
2	**2/4**	32/32	16/32	32/32	>128	>128	>128	>128
4	**4/8**	**2/4**	**4/4**	**4/8**	16/32	16/32	16/16	**8/16**
4b	**8/8**	**4/4**	**4/4**	**4/8**	32/64	32/64	32/32	32/64
5	**2/2**	**4/4**	**4/4**	**2/4**	**8/8**	**8/8**	**8/8**	**8/8**
5b	**2/2**	**4/8**	**4/4**	**2/4**	16/32	**8/32**	**8/32**	**8/8**
6	**4/8**	-	-	-	-	-	-	-
7	**4/8**	**2/4**	**2/4**	**4/4**	**8/8**	**8/8**	**8/8**	**8/8**
9	**4/8**	32/32	32/32	32/32	>128	>128	>128	>128
10	**4/8**	32/32	16/16	16/16	>128	>128	>128	>128
11	**2/2**	32/32	**2/4**	**2/4**	**8/8**	**8/32**	**8/16**	**8/8**

^a^ Minimum Inhibitory Concentrations and Minimum Bactericidal Concentrations are reported for each compound, unless no significant direct antibacterial activity could be detected (MIC, >128 µg/mL); MIC between 2 and 8 µg/mL are highlighted in bold, with a green background. -, not determined. The resistance determinants or phenotypes (see Table 4 for details) are indicated between parentheses for each isolate (MRSA, methicillin resistant *S. aureus*; OXA-23, OXA-24, and OXA-51-like are class D carbapenem-hydrolyzing β-lactamases; IMP-1, VIM-1, and VIM-2 are metallo-β-lactamases).

**Table 4:** Clinical isolates used in this study and their phenotype of resistance to antibiotics.

Clinical Isolate	Resistance Phenotype ^a^
*Staphylococcus aureus* ATCC 43300	PEN (methicillin-resistant)
*Acinetobacter baumannii* SI-12	PEN, CARB (*bla*_OXA-23_^+^), AG, FQ, SXT
*Acinetobacter baumannii* SI-310	PEN, CARB (*bla*_OXA-24_^+^), AZT, AG, FQ, SXT, COL
*Acinetobacter baumannii* SI-648	PEN, CARB (*bla*_OXA-23_^+^ + hyperproduction of OXA-51-like enzyme), AG, FQ, SXT
*Pseudomonas aeruginosa* VR-143/97	PEN, ES-CEPH, CARB (*bla*_VIM-1_^+^), AZT, AG, FQ
*Pseudomonas aeruginosa* VA-182/00	PEN, ES-CEPH, CARB (*bla*_VIM-2_^+^), AZT, AG, FQ
*Pseudomonas aeruginosa* 101/1477	PEN, ES-CEPH, CARB (*bla*_IMP-1_^+^), AG
*Pseudomonas aeruginosa* 506/99	PEN, ES-CEPH, CARB (*bla*_VIM-2_^+^), AG

^a^ PEN, penicillins; ES-CEPH, expended-spectrum cephalosporins; CARB, carbapenems (the carbapenemase-encoding gene(s) found in the isolate is (are) indicated between parentheses), AZT, aztreonam (a monobactam); AG, aminoglycosides; FQ, fluoroquinolones; SXT, trimethoprim-sulfamethoxazole; FOS, fosfomycin; COL, colistin.

## Data Availability

Data supporting reported results can be obtained from the authors.

## Supplementary Materials

The Supporting Materials can be downloaded at: https://www.mdpi.com/article/10.3390/ijms24097997/s1.
