# Supplementary material for: Peptaibol Analogs Show Potent Antibacterial Activity against Multidrug Resistant Opportunistic Pathogens
Source: Int J Mol Sci. 2023 Apr 28;24(9):7997. doi: 10.3390/ijms24097997 (PMC10178204; doi:10.3390/ijms24097997)
Supplement: Supplementary file 1 [file ijms-24-07997-s001.zip › ijms-2358466-supplementary.pdf]

SUPPORTING MATERIAL

Peptaibol Analogs Show Potent Antibacterial Activity against Multidrug Resistant Opportunistic Pathogens

Chiara Dalla Torre [1], Filomena Sannio [2], Mattia Battistella [1], Jean-Denis Docquier [2,3,4] and Marta De Zotti [1,*]

[1] Department of Chemical Sciences, University of Padova, Via Marzolo 1, I-35131 Padova, Italy; chiara.dallatorre.2@studenti.unipd.it (C.D.T.); mattia.battistella.1@studenti.unipd.it (M.B.)
[2] Dipartimento di Biotecnologie Mediche, University of Siena, Viale Bracci 16, I-53100 Siena, Italy; filomena.sannio@unisi.it (F.S.); jddocquier@unisi.it (J.-D.D.)
[3] Lead Discovery Siena s.r.l., Via Fiorentina 1, I-53100 Siena, Italy
[4] Laboratoire de Bactériologie Moléculaire, Centre d'Ingénierie des Protéines—UR InBioS, University of Liège, Allée du six Août 11, B-4000 Liège, Belgium
* Correspondence: marta.dezotti@unipd.it; Tel.: +39-049-827-5280

TABLE OF CONTENTS:

1. Figures S1–S12: HPLC and ESI-HRMS spectra for the new sequences 13–18
2. Tables S1–S2: minimum bactericidal concentrations (MBCs),
   Figure S13: hemolysis zones

1. HPLC and ESI-HRMS spectra for the new sequences 13–18

Sequences of the new peptides synthesized in this work.[a]

13	UKKLL	1-Oct-Aib-Lys-Lys-Leu-Leu-NH_2
14	LUKI	1-Oct-Leu-Aib-Lys-Ile-NH_2
15	UKLL	1-Oct-Aib-Lys-Leu-Leu-NH_2
16	LUKL	1-Oct-Leu-Aib-Lys-Leu-NH_2
17	KLUL	1-Oct-Lys-Leu-Aib-Leu-NH_2
18	LKUL	1-Oct-Leu-Lys-Aib-Leu-NH_2

[a]U, Aib: α-aminoisobutyric acid; 1-Oct: 1-octanoyl.

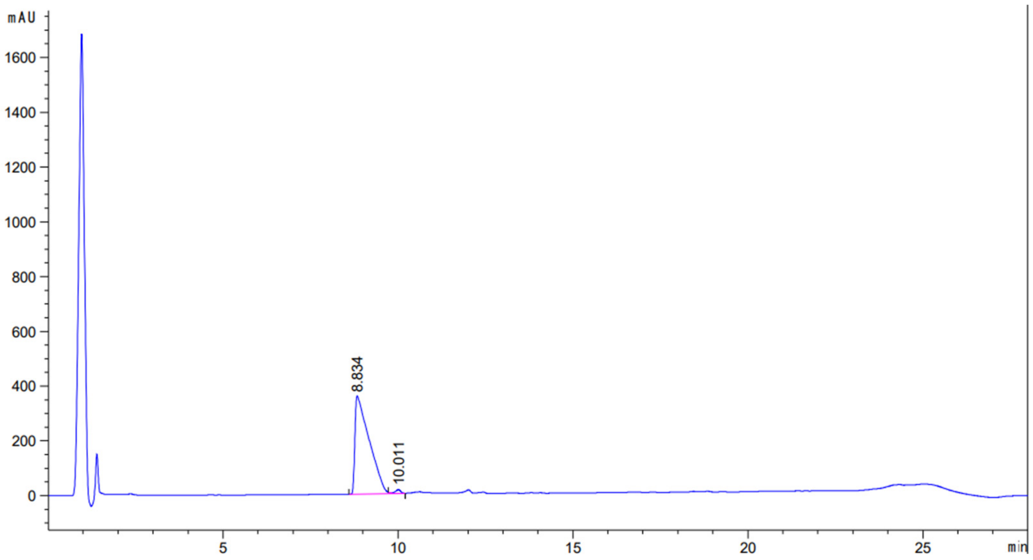

Figure S1. HPLC profile acquired for peptide **13** UKKLL. Agilent Poroshell 120 4μm EC-C18–column. Gradient: 20-80 %B in 18 min – Flow: 1mL/min - Detection UV: 220 nm - Rt=8.834 min; Purity=98%

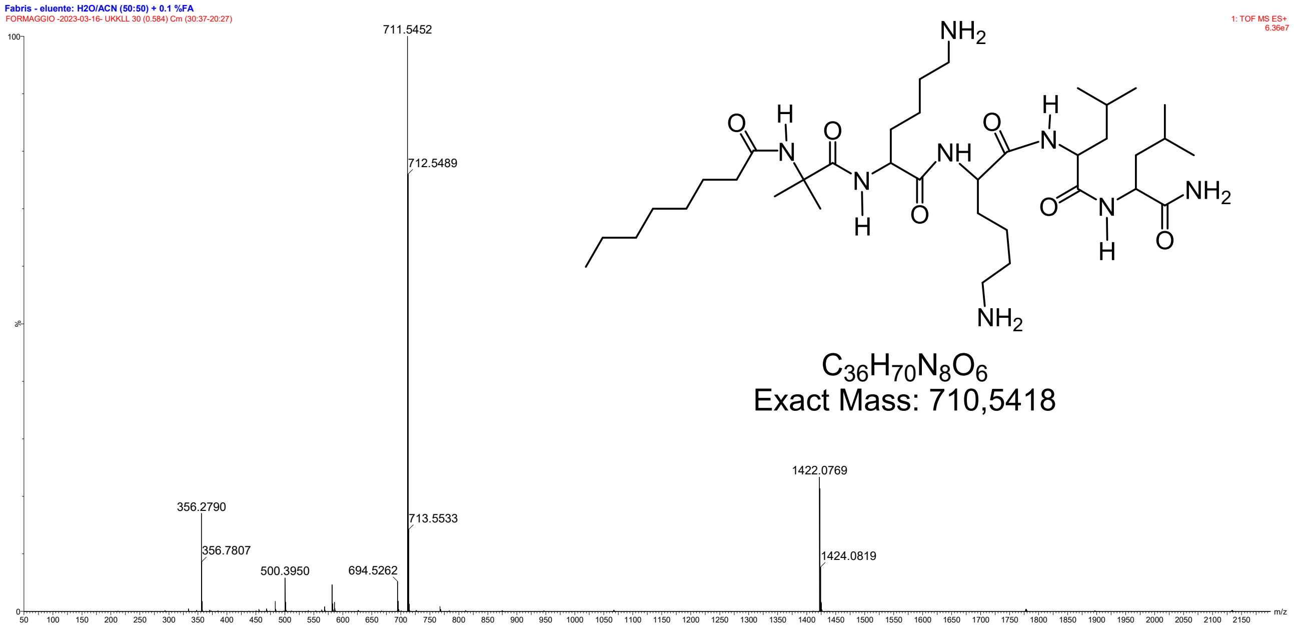

Figure S2. ESI-HRMS spectrum for peptide **13**. $[M+H]^+_{found}$ = 711.5452 m/z; dimer $[2M+H]^+_{found}$ = 1422.0769 m/z; $[M+2H]^{++}_{found}$ = 356.2790 m/z.

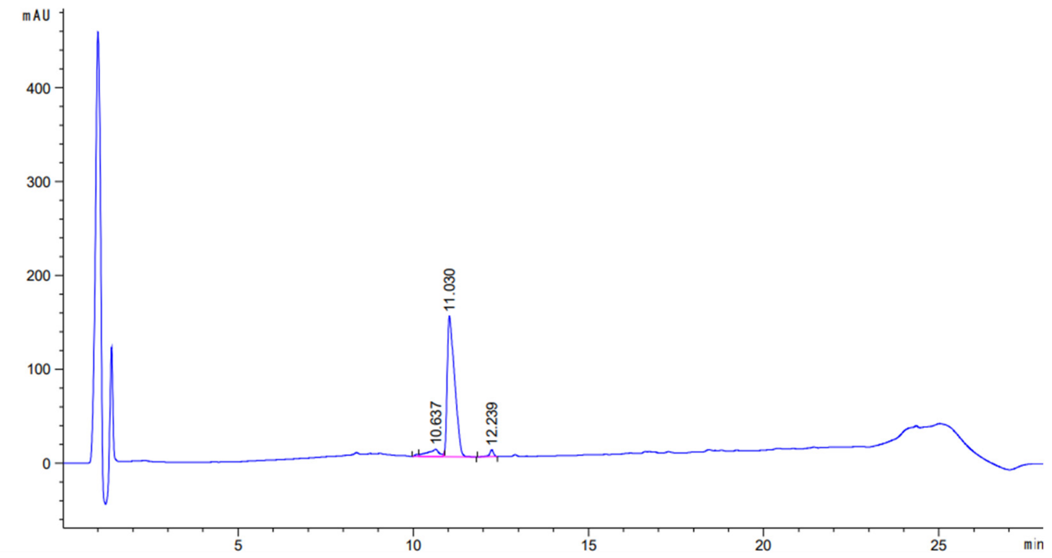

Figure S3. HPLC profile acquired for peptide **14** LUKI. Agilent Poroshell 120 4μm EC-C_{18}–column; Gradient: 20-80%B in 18 min, flow: 1 mL/min - Detection UV: 220 nm, Rt =11.030 min. Purity 91%

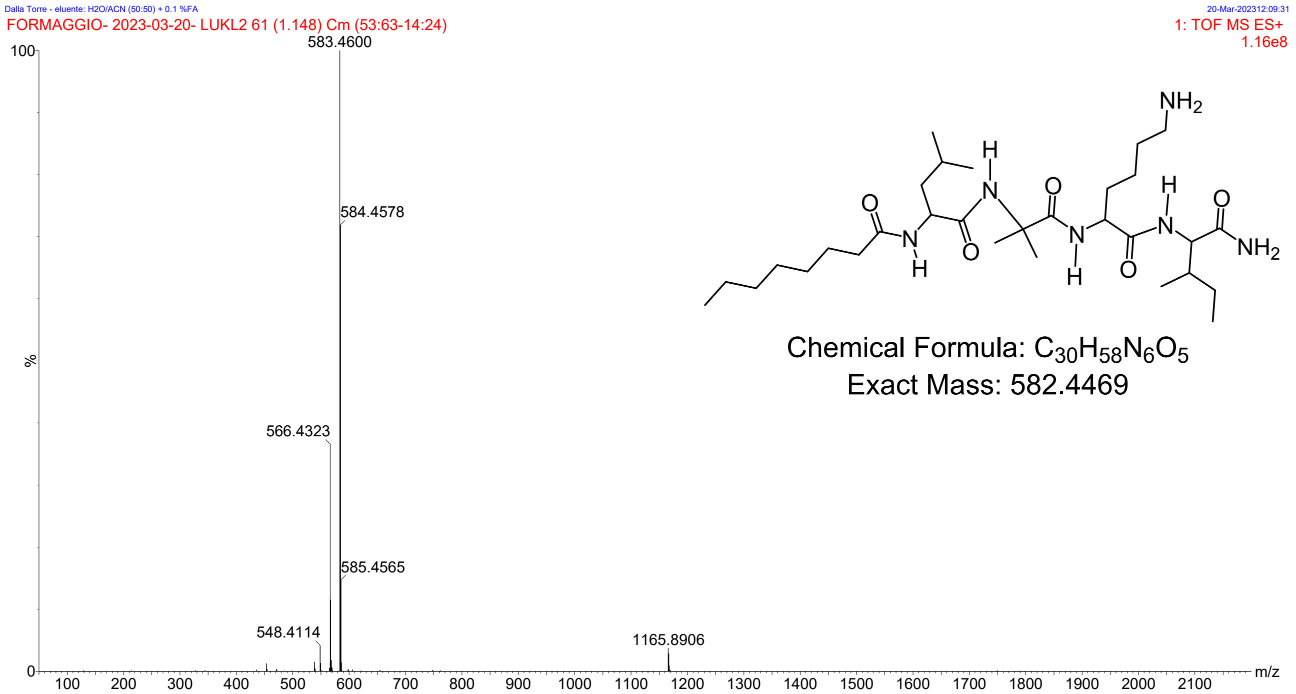

Figure S4. ESI-HRMS spectrum for peptide **14**. $[M+H]^+_{found}$ = 583.4600 m/z; fragment $[M-NH_2]^+_{found}$ = 566.4323 m/z; dimer $[2M+H]^+_{found}$ = 1165.8906 m/z.

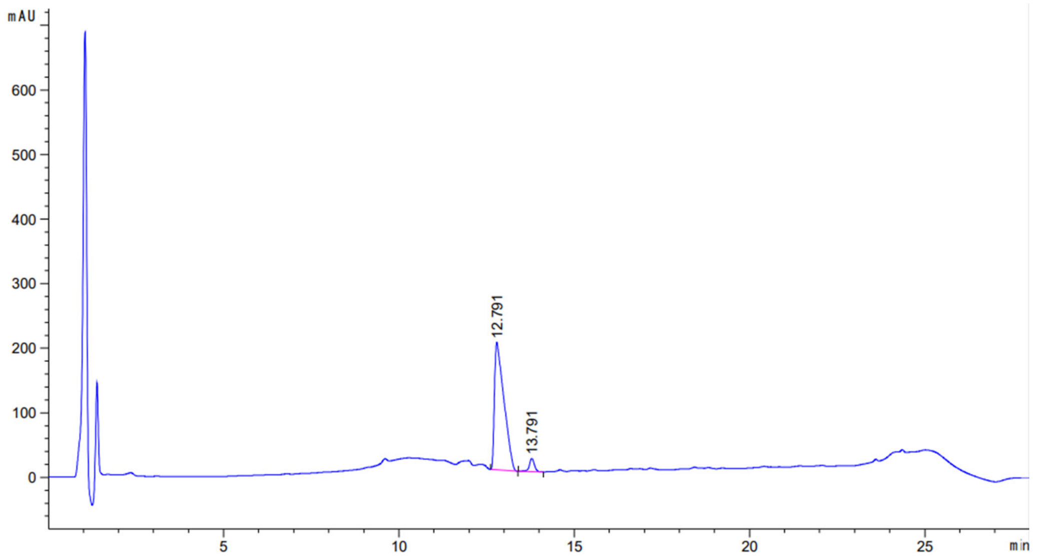

Figure S5. HPLC profile acquired for peptide **15** UKLL. Agilent Poroshell 120 4μm EC-C18–column. Gradient: 20-80 %B in 18 min – Flow: 1mL/min - Detection UV: 220 nm - Rt=12.791 min; Purity=95%

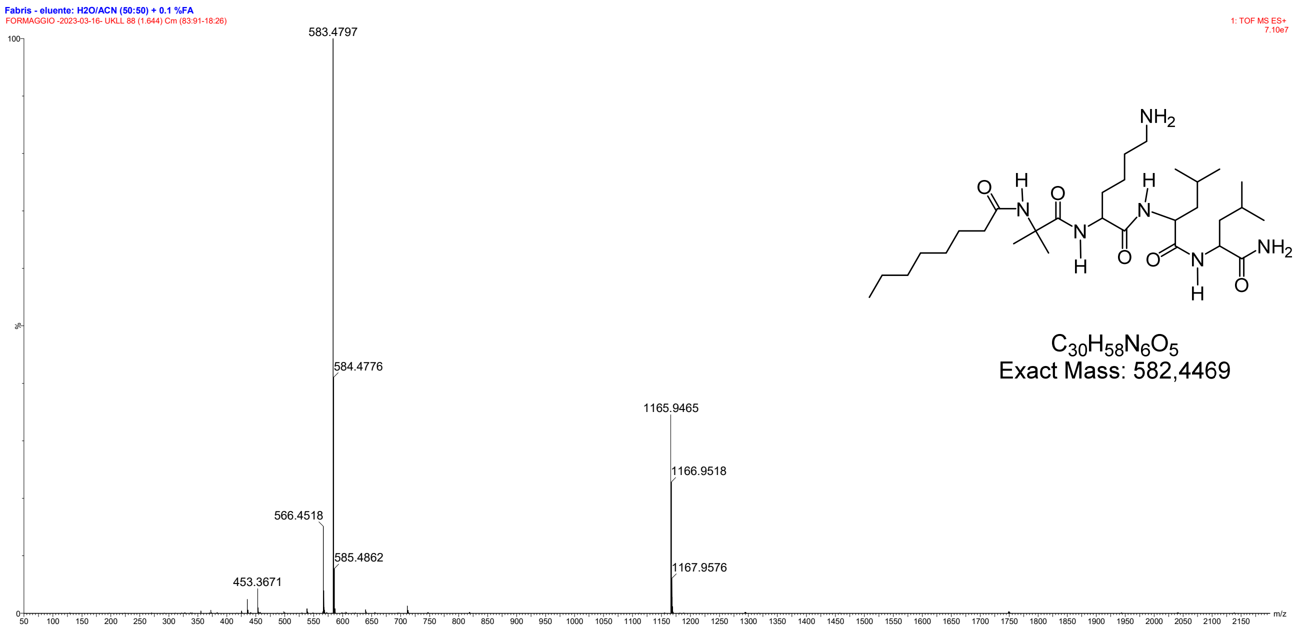

Figure S6. ESI-HRMS spectrum for peptide **15** UKLL. $[M+H]^+_{found}$ = 583.4797 m/z; $[2M+H]^+_{found}$ = 1165.9465 m/z; fragment $[M-NH_2]^+_{found}$ = 566.4518 m/z.

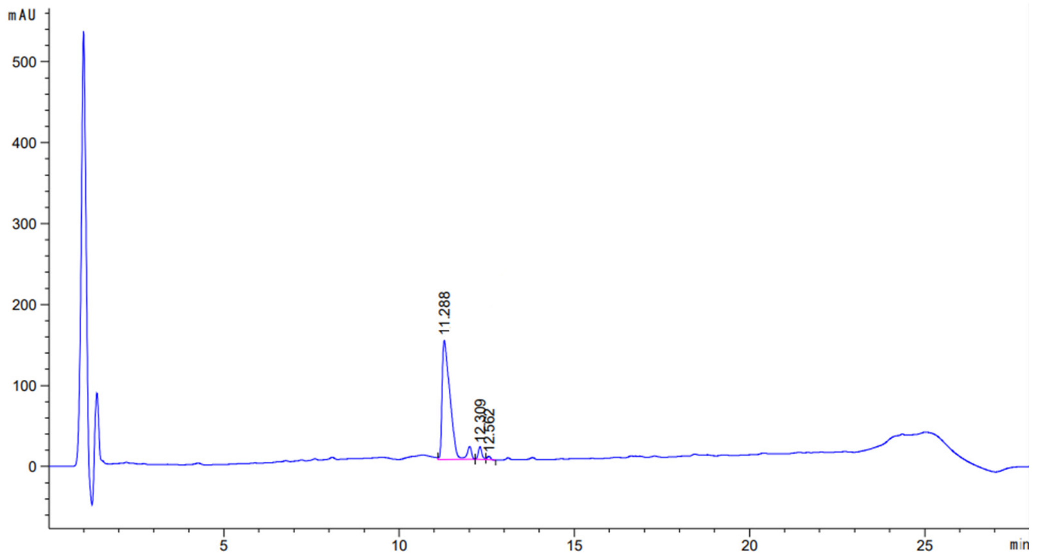

Figure S7. HPLC profile acquired for peptide **16** LUKL. Agilent Poroshell 120 4μm EC-C18–column. Gradient: 20-80 %B in 18 min – Flow: 1mL/min - Detection UV: 220 nm - Rt =11.288 min; Purity=94%

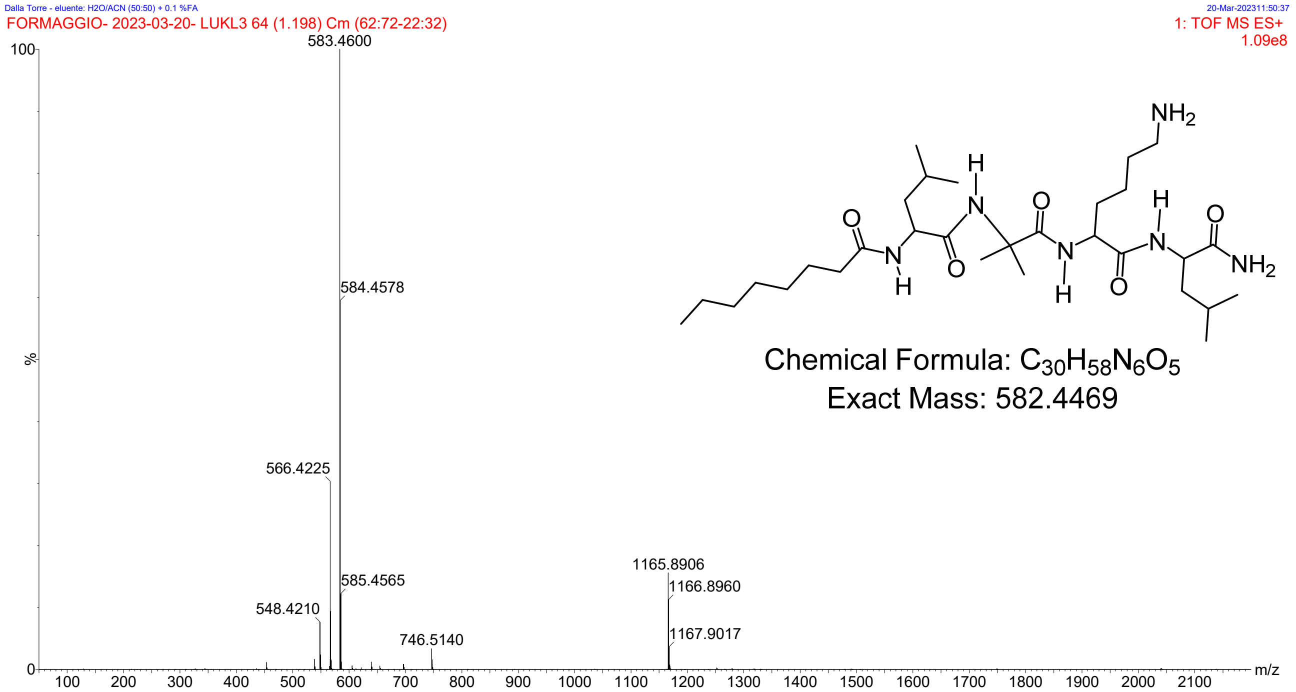

Figure S8. ESI-HRMS spectrum for peptide **16.** $[M+H]^{+}_{found}$ = 583.4600 m/z; $[2M+H]^{+}_{found}$ = 1165.8906 m/z; fragment $[M-NH_2]^{+}_{found}$ = 566.4225 m/z.

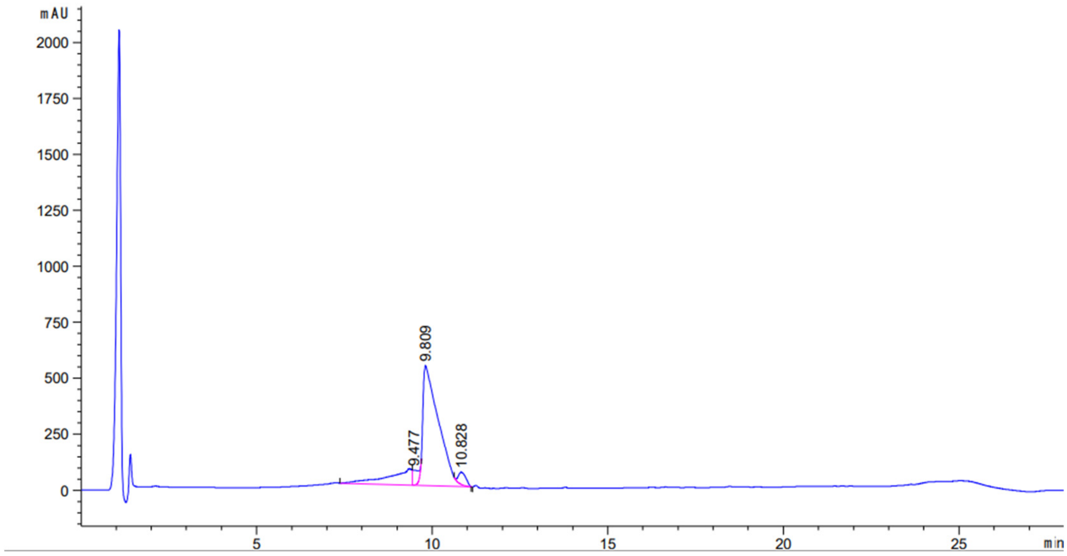

Figure S9. HPLC profile acquired for peptide **17** KLUL. Agilent Poroshell 120 4µm EC-C_{18}–column. Gradient: 20-80 %B in 18 min – Flow: 1mL/min - Detection UV: 220 nm - Rt=9.809 min; Purity=92%

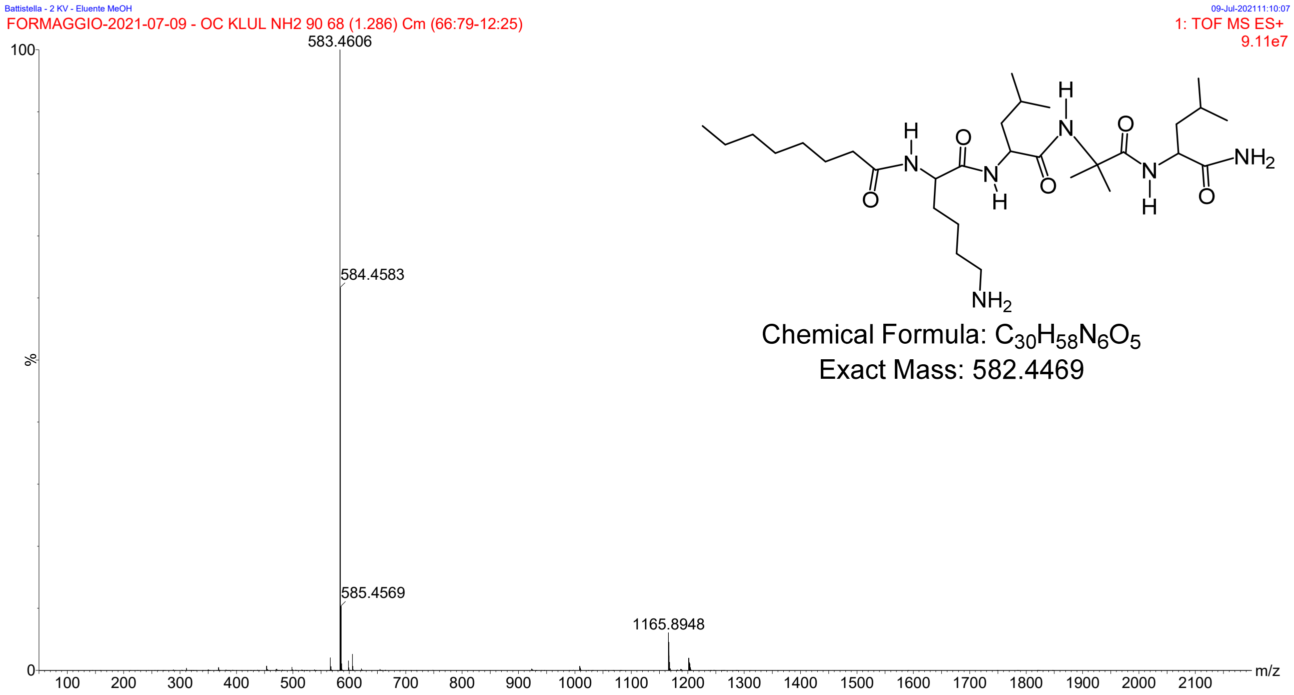

Figure S10. ESI-HRMS spectrum for peptide **17**. $[M+H]^+_{found}$ = 583.4606 m/z; $[2M+H]^+_{found}$ = 1165.8948 m/z.

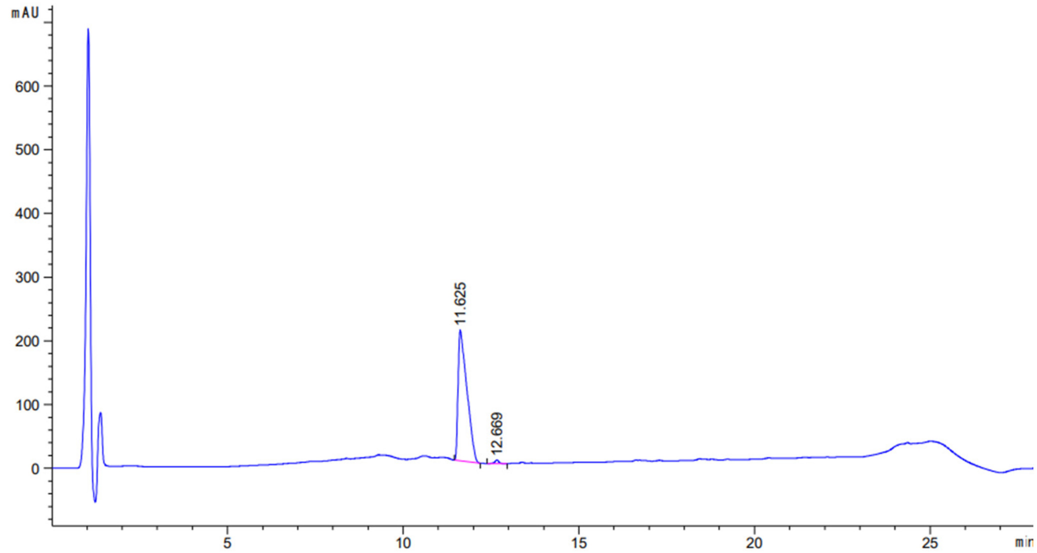

Figure S11. HPLC profile acquired for peptide **18** LKUL. Agilent Poroshell 120 4μm EC-C_{18}–column. Gradient: 20-80 %B in 18 min – Flow: 1mL/min - Detection UV: 220 nm - Rt =11.625 min; Purity=98%

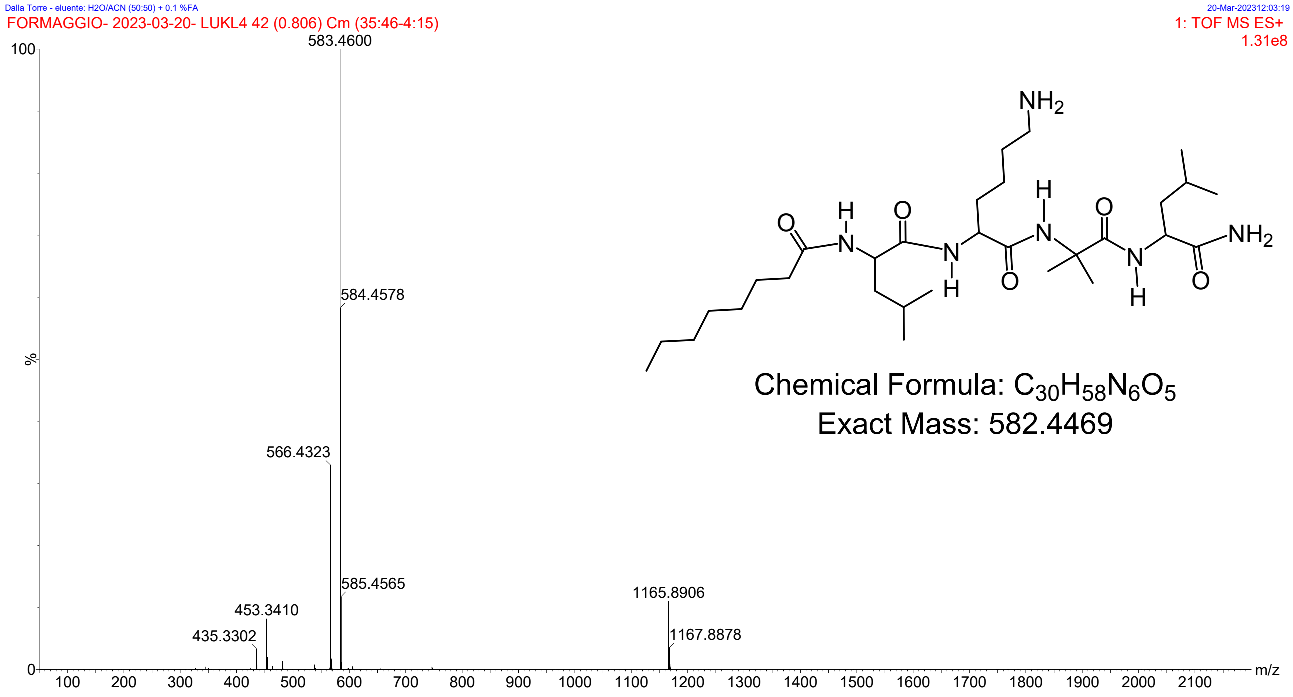

Figure S12. ESI-HRMS spectrum for peptide **18**. $[M+H]^{+}_{found}$ = 583.4600 m/z; fragment $[M-NH_2]^{+}_{found}$ = 566.4323 m/z; dimer $[2M+H]^{+}_{found}$ = 1165.8906 m/z.

Table S1. *In vitro* antibacterial activity (MIC and MBC) of compounds on reference strains.

Peptide name	MIC and MBC (μg/ml)[a]							
	Bsu	Efa	Spy	Sau	Eco	Kpn	Aba	Pae
**0 Tric-Lol**	16/32	16/32	16/16	>128	>128	>128	>128	>128
**1 [K^{2,5}]-Lol**	**4/16**	**2/2**	**2/4**	16	32/32	>128	**4/16**	16
**2 [K^{2}]-NH$_2$**	**4/16**	**4/8**	**4/4**	**8/8**	>128	64/>128	16/16	>128
**3 [Leu^{4}]-NH$_2$**	>128	>128	>128	>128	>128	>128	>128	>128
**4 [K^{5,6}]-Lol**	**2/8**	**2/8**	**2/2**	16/16	16/>16	16/32	**2/2**	**8/16**
**4b [K^{5,6}]-NH$_2$**	**2/8**	**4/8**	**2/8**	16/16	16/>16	32/>16	**4/4**	16/>16
**5 [K^{2,5,9}]-Lol**	**8/16**	**4/8**	**4/4**	16/16	**8/>16**	16/16	**4/16**	**8/>16**
**5b [K^{2,5,9}]-NH$_2$**	**2/16**	**2/16**	**2/32**	**8/32**	32/128	32/128	**4/32**	16/128
**6 [K^{5},U^{6}]-Lol**	**8/16**	**8/8**	**8/8**	**8/16**	>128	>128	>128	>128
**6b [K^{5},U^{6}]-NH$_2$**	128/128	128/128	**8/32**	128/128	>128	>128	>128	>128
**7 [K^{2,5,6,9}]-Lol**	**2/16**	**4/8**	**2/8**	16/32	16/16	16/16	**2/16**	**8/>16**
**8 [Api^{8}]-NH$_2$**	32/32	16/16	16/16	>128	>128	>128	>128	>128
**9 [K^{9}]-Lol**	**4/16**	**4/4**	16/32	16/16	>128	>128	**8/8**	>128
**10 [K^{6}]-Lol**	**4/16**	**4/16**	**4/16**	**8/16**	64/128	16/64	16/>128	**8/128**
**10b [K^{6}]-NH$_2$**	**4/16**	**4/8**	**4/16**	32/32	>128	>128	16/16	>128
**11 [K^{2,5,6}]-NH$_2$**	**2/16**	**4/32**	**4/16**	16	16/128	>128	**2/2**	>128
**12 [4-11]**	4/16	32/32	16/16	16/16	16/64	64/>128	32/32	64/>128
**13 UKKLL**	32/64	>128	>128	>128	>128	>128	>128	>128
**14 LUKI**	>128	>128	>128	>128	>128	>128	>128	>128
**15 UKLL**	16/32	32/32	16/16	64/>128	64/>128	64/>128	64/128	64/>128
**16 LUKL**	>128	>128	>128	>128	>128	>128	>128	>128
**17 KLUL**	>128	>128	>128	>128	>128	>128	>128	>128
**18 LKUL**	128/>128	>128	128	>128	>128	>128	>128	>128

[a]Gram positive: *Bacillus subtilis* ATCC 6633 (Bsu), *Enterococcus faecalis* ATCC 29212 (Efa), *Streptococcus pyogenes* ATCC 12344 (Spy), *Staphylococcus aureus* ATCC 25923 (Sau); Gram negative: *Escherichia coli* CCUGT (Eco), *Klebsiella pneumoniae* ATCC 13833 (Kpn), *Acinetobacter baumannii* ATCC 17978 (Aba), *Pseudomonas aeruginosa* ATCC 27853 (Pae).

Table S2. *In vitro* antibacterial activity (MIC) of trichogin GA IV and its analogs against clinical isolates of *S.aureus*, *A. baumannii*, and *P. aeruginosa*.

Peptide No.	MIC/MBC (μg/ml)[a]							
	S.aureus ATCC 43300 (MRSA)	*A. baumannii SI-12 (OXA-23)*	*A. baumannii SI-648 (OXA-23, OXA-51-like (ISAba))*	*A. baumannii SI-310 (OXA-24)*	*P. aeruginosa VR-143/97 (VIM-1)*	*P. aeruginosa VA-182/00 (VIM-2)*	*P. aeruginosa 101/1477 (IMP-1)*	*P. aeruginosa 506/99 (IMP-2)*
**1 [K^{2,5}]-Lol**	2/2	4/8	8/16	4/4	32/32	16/16	8/16	16/32
**2 [K^{2}]-NH$_2$***	2/4	32/32	16/32	32/32	>128	>128	>128	>128
**4 [K^{5,6}]-Lol**	4/8	2/4	4/4	4/8	16/32	16/32	8/16	16/16
**4b [K^{5,6}]-NH$_2$**	8/8	4/4	4/4	4/8	32/64	32/64	32/64	32/32
**5 [K^{2,5,9}]-Lol**	2/2	4/4	4/4	2/4	8/8	8/8	8/8	8/8
**5b [K^{2,5,9}]-NH$_2$**	2/2	4/8	4/4	2/4	16/32	8/32	8/8	8/32
**6 [K^{5},U^{6}]-Lol**	4/8	-	-	-	-	-	-	-
**7 [K^{2,5,6,9}]-Lol**	4/8	2/4	2/4	4/4	8/8	8/8	8/8	8/8
**9 [K^{9}]-Lol**	4/8	32/32	32/32	32/32	>128	>128	>128	>128
**10 [K^{6}]-Lol**	4/8	32/32	16/16	16/16	>128	>128	>128	>128
**11 [K^{2,5,6}]-NH$_2$***	2/2	32/32	2/4	2/4	8/8	8/32	8/8	8/16

[a] MIC between 2 and 8 μg/mL are highlighted with a green background. -, not determined.

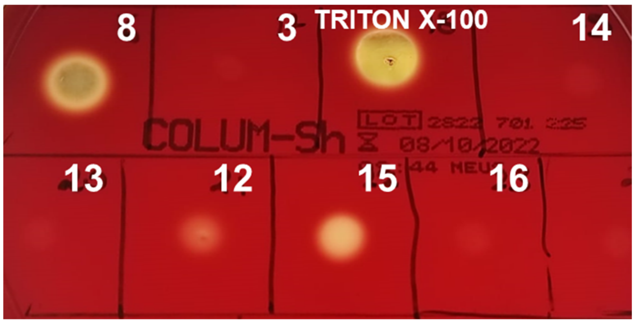

Figure S13. Implementation of the hemolysis test as described in Bechlars *et al*. [58]. After spotting 2 μL (50 μg) of each compound at a random location on the blood agar plate and incubation at 35 ± 2 °C for 24 h, the plates were manually inspected for the presence of hemolysis zones. Triton X-100 and DMSO were used as the positive and negative controls, respectively. Results were interpreted as follows: compounds 3, 13, 14 and 16, no hemolysis detected; compound 12, limited hemolysis (indicated as -* in Table 2); compounds 8 and 15, hemolytic activity (the average value of the diameters of the hemolysis zone measured in individual experiments is reported in Table 2).
